# Protection from COVID-19 with a VSV-based vaccine expressing the spike and nucleocapsid proteins

**DOI:** 10.3389/fimmu.2022.1025500

**Published:** 2022-10-24

**Authors:** Kyle L. O’Donnell, Tylisha Gourdine, Paige Fletcher, Chad S. Clancy, Andrea Marzi

**Affiliations:** ^1^ Laboratory of Virology, Division of Intramural Research, National Institute of Allergy and Infectious Diseases, National Institutes of Health, Hamilton, MT, United States; ^2^ Rocky Mountain Veterinary Branch, Division of Intramural Research, National Institute of Allergy and Infectious Diseases, National Institutes of Health, Hamilton, MT, United States

**Keywords:** severe acute respiratory syndrome coronavirus 2, SARS-CoV-2, intranasal vaccination, vesicular stomatitis virus, hamster model

## Abstract

Successful vaccine efforts countering the COVID-19 pandemic are centralized around the severe acute respiratory syndrome coronavirus 2 (SARS-CoV-2) spike (S) protein as viral antigen and have greatly reduced the morbidity and mortality associated with COVID-19. Since the start of this pandemic, SARS-CoV-2 has evolved resulting in new variants of concern (VOC) challenging the vaccine-established immunologic memory. We show that vaccination with a vesicular stomatitis virus (VSV)-based vaccine expressing the SARS-CoV-2 S plus the conserved nucleocapsid (N) protein was protective in a hamster challenge model when a single dose was administered 28 or 10 days prior to challenge, respectively. In this study, only intranasal vaccination resulted in protection against challenge with multiple VOC highlighting that the addition of the N protein indeed improved protective efficacy. This data demonstrates the ability of a VSV-based dual-antigen vaccine to reduce viral shedding and protect from disease caused by SARS-CoV-2 VOC.

## Introduction

Since December 2019, the severe acute respiratory syndrome coronavirus 2 (SARS-CoV-2) has spread rapidly throughout the globe causing over 580 million infections and 6.4 million deaths (1). The CoV disease 2019 (COVID-19) pandemic is placing a considerable burden on health care systems worldwide and has socioeconomic impacts due to the lack of control of viral spread. Numerous SARS-CoV-2 vaccines have shown efficacy in extensive phase 3 clinical trials. Several of these vaccines have been licensed for human use including large-scale vaccination campaigns. Today, most vaccines use the surface spike (S) protein of the original Wuhan isolate as the primary vaccine antigen (2–4). Emerging SARS-CoV-2 variants have shown increasing degrees in divergence of the S protein challenging vaccine efficacy (5, 6). In contrast to the S protein, the nucleocapsid (N) protein is a highly conserved antigen with 90% amino acid homology to SARS-CoV-1 and fewer mutations in emerging SARS-CoV-2 variants of concern (VOC) over time (7–10). The N protein is highly immunogenic, eliciting both a humoral and cellular response during infection (11, 12). Unlike neutralizing antibody responses, T cell immunity is not limited to surface antigens and a potent cytotoxic T cell response has been reported upon stimulation with N-specific peptide pools (13). Within the N protein, strong T cell epitopes have been identified (13–17). The resulting N-specific T cell response is not only specific for SARS-CoV-2 but has the potential for cross-reactivity to other seasonal coronaviruses such as OC43, NL63, and HKU-1 (15). T cell immunity may be less vulnerable to immune selection pressure and viral escape, thus maintaining vaccine efficacy across a continued emergence of viral variants including VOC. Therefore, the introduction of a second antigen to an existing vaccine may stimulate cytotoxic T cells and increase the breadth of the antibody response to maintain vaccine efficacy across SARS-CoV-2 variants.

The recombinant vesicular stomatitis virus (VSV)-based vaccine platform has been successfully used for multiple viral pathogens such as Ebola virus (EBOV), Nipah virus, and Lassa virus expressing the viral surface glycoprotein (GP) as the vaccine antigen (18–20). Previously, we demonstrated the efficacy of intramuscular (IM) and intranasal (IN) vaccination of hamsters with a VSV vaccine expressing the S protein and the EBOV GP (VSV-SARS2-EBOV) or a vaccine expressing only the S protein (VSV-SARS2). The studies resulted in superior protective efficacy after IN vaccination from challenge with the SARS-CoV-2 ancestral, Alpha, Beta, and Delta variants (21). Here, we updated our existing VSV-SARS2 vaccine by co-expressing the SARS-CoV-2 N and S proteins (VSV-SARS2 N+S). VSV-based vaccines have been shown to elicit a robust humoral immune response with limited T cell involvement to the encoded antigen(s) after a single immunization. By incorporating a strong T cell antigen into our existing vaccine, we aimed to strengthen the cellular response after vaccination. The stimulation of a more balanced cellular and humoral immune response coupled with unique attributes - robust immune stimulation and short time to immunity - make VSV an attractive viral vector vaccine platform for SARS-CoV-2.

## Materials and methods

### Ethics statement

Infectious work with SARS-CoV-2 was performed in the containment laboratories at the Rocky Mountain Laboratories (RML), Division of Intramural Research, National Institute of Allergy and Infectious Diseases, National Institutes of Health. RML is an institution accredited by the Association for Assessment and Accreditation of Laboratory Animal Care International. The studies were approved by the RML Animal Care and Use Committee. Animal work was performed in strict accordance with the recommendations described in the Guide for the Care and Use of Laboratory Animals of the National Institute of Health, the Office of Animal Welfare and the Animal Welfare Act, United States Department of Agriculture. All procedures followed standard operating procedures (SOPs) approved by the RML Institutional Biosafety Committee (IBC). Procedures were conducted in animals anesthetized by trained personnel. All efforts were made to ameliorate animal welfare and minimize animal suffering; food and water were provided *ad libitum*.

### Cells and viruses

Vero E6 cells (mycoplasma negative) were grown at 37°C and 5% CO_2_ in Dulbecco’s modified Eagle’s medium (DMEM) (Sigma-Aldrich, St. Louis, MO) containing 10% fetal bovine serum (FBS) (Wisent Inc., St. Bruno, Canada), 2 mM L-glutamine, 50 U/mL penicillin, and 50 μg/mL streptomycin (all Thermo Fisher Scientific, Waltham, MA). Baby hamster kidney cells expressing T7 polymerase (BHK-T7) were grown at 37°C and 5% CO_2_ in minimum essential medium (MEM) containing 10% tryptose phosphate broth (both Thermo Fisher Scientific), 5% FBS, 2 mM L-glutamine, 50 U/mL penicillin, and 50 μg/mL streptomycin. SARS-CoV-2 Alpha variant (hCOV_19/England/204820464/2020), Beta variant (hCoV-19/South African/KRISP-K005325/2020), and Delta variant (nCoV-19/USA/KY-CDC-2-4242084/2021) were used for the hamster challenge studies and neutralization testing. Omicron variant BA.2 (SCV2/USA/MD-HP24556/2022) was used for neutralization testing. All viral stocks were grown and titered on Vero E6 cells and their sequence was confirmed.

### Generation of VSV-based vaccine candidates

The SARS-CoV-2 N open reading frame (ORF) was PCR-amplified from an expression plasmid encoding the codon-optimized (human) gene based on GenBank accession number MN908947. Full-length SARS-CoV-2 N was cloned into the pATX-VSV-SARS2 plasmid upstream of the SARS-CoV-2 S protein, resulting in VSV-SARS2-N+S (**Figure S1A**) following a previously successful strategy (21, 22). The replication-competent recombinant VSV was recovered from plasmid in BHK-T7 cells as described previously; VSV-SARS2-N+S was propagated on Vero E6 cells (23). The complete sequence of the viruses was confirmed by Sanger sequencing. The titer of the virus stocks was determined using a standard plaque assay on Vero E6 cells.

### Growth kinetics

Vero E6 cells were grown to confluency in a 12-well plate and infected in triplicate with VSVwt, VSV-EBOV, or VSV-SARS2-N+S at a multiplicity of infection of 0.01. After 1 h incubation at 37°C, cells were washed three times with plain DMEM, and covered with DMEM containing 2% FBS. Supernatant samples were collected at 0, 12, 24, 48, 72, and 96 hours post-infection and stored at −80 °C. The titer of the supernatant samples was determined performing a median tissue infectious dose (TCID_50_) assay on Vero E6 cells as previously described (23).

### Western blot analysis

Supernatant or infected Vero E6 cell samples containing VSV were mixed with sodium dodecyl sulfate-polyacrylamide (SDS) gel electrophoresis sample buffer containing 20% β-mercaptoethanol and heated to 99 °C for 10 min. Analysis of the samples was performed as described elsewhere (24). SARS-CoV-2 N or S expression was detected using anti-SARS-CoV-2 N or anti-SARS-CoV-2 S (1:1000; Sino Biological, Chesterbrook, PA, USA) antibody. Wildtype VSV samples served as a negative control for the N- and S-specific blots while a cell lysate of SARS-CoV-2 N was used as a positive control for the N-specific blot and negative control for the S-specific blot.

### Animal study

Ninety female Syrian golden hamsters (5-8 weeks of age) were used in this study. The hamsters were randomly divided into five vaccine groups (n=12/group) and vaccinated 28 or 10 days prior to challenge with a single vaccine dose of 1x10^5^ PFU of VSV-SARS2-N+S by the IM (n=6) or IN (n=6) route. Control animals received the same dose of a control vaccine (VSV-EBOV) by either the IM (n=3) or IN (n=3) route; data were combined into one control group. On day 0, all hamsters were challenged with SARS-CoV-2 as previously described (25). On day 4 post challenge, all hamsters were euthanized for sample collection.

### RNA extraction, RT-qPCR and virus titration

RNA from samples were extracted using the QIAamp Viral RNA Mini Kit (Qiagen, Hilden, Germany) or the RNeasy Mini Kit (Qiagen) according to manufacturer specifications. One step RT-qPCR for genomic viral RNA was performed as described previously (26). Five μL of each RNA extract were run alongside dilutions of SARS-CoV-2 standards with a known concentration of RNA copies. Virus titrations were performed, and titers calculated as described previously (27)

### Enzyme-linked immunosorbent assay and virus neutralization

Serum samples from SARS-CoV-2-challenged hamsters were inactivated by γ-irradiation and used in BSL2 according to IBC-approved SOPs. For the ELISA, Nunc Maxisorp Immuno plates (Thermo Fisher Scientific) were coated with 50 μl of 1 μg/mL of recombinant SARS-CoV-2 S (S1+S2) or SARS-CoV-2 N antigen (Sino Biological, Chesterbrook, PA, USA) and ELISA was performed as described previously (24). The optical density (OD) at 405 nm was measured using a GloMax^®^ explorer (Promega). The OD values were normalized to the baseline samples obtained with naïve hamster serum and the cutoff value was set as the mean OD plus three times the standard deviation of the blank. Virus neutralization assay was performed as described previously (24).

### Histology and immunohistochemistry

Hamster lung samples were fixed in 10% neutral buffered formalin with two changes, for a minimum of 7 days. The lung sections were then processed as previously described (27). All tissue slides were evaluated blindly by a board-certified veterinary pathologist.

### Statistical analyses

All statistical analysis was performed in Prism 8 (GraphPad). Serology, cellular responses, and virology were examined by Mann-Whitney test. Vaccine growth kinetics were examined using one-way ANOVA with Tukey’s multiple comparisons to evaluate statistical significance at all timepoints. Statistically significant differences are indicated as follows: p<0.0001 (****), p<0.001 (***), p<0.01 (**) and p<0.05 (*).

## Results

### Vaccine construction and characterization

We previously constructed a VSV full-length plasmid encoding the SARS-CoV-2 S protein. Here, we used this plasmid as the parental vector to construct the SARS-CoV-2 vaccine expressing both N and S proteins. To this end, we introduced the SARS-CoV-2 N upstream of the S protein in the existing vector (**Figure S1A**). The construct was recovered following a previously established protocol (23). Antigen expression was confirmed for both proteins by Western blot analysis of the supernatant or cell lysate of infected cells (**Figure S1B**). Viral growth kinetics of the VSV-SARS2-N+S compared to VSV-EBOV and the parental VSV-SARS2 resulted in delayed growth of the VSV-SARS2-N+S vaccine and a lower endpoint titer similar to the parental VSV-SARS2 (**Figure S1C**).

### Intranasal administration of VSV-SARS2-N+S protects hamster from COVID-19

**Figure 1:**
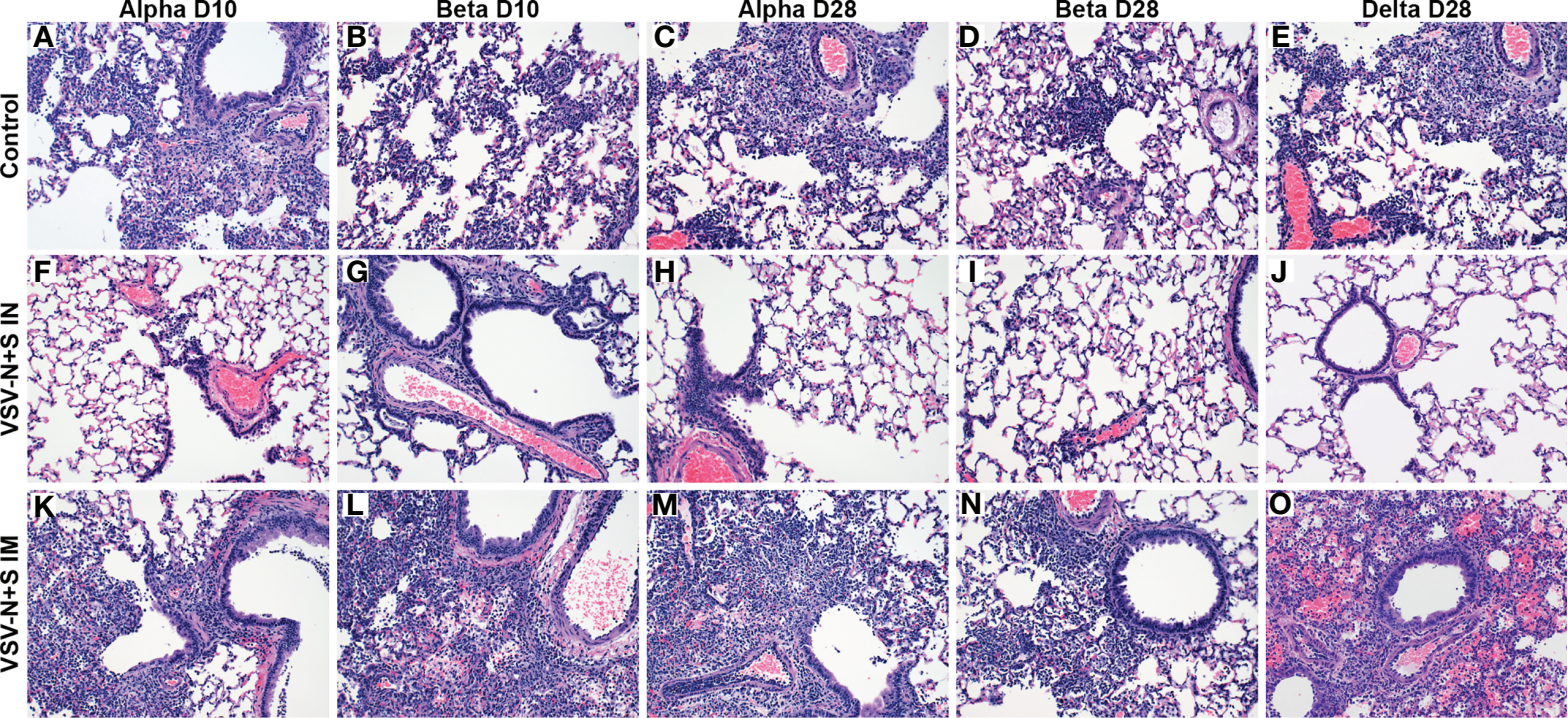
Hamster lung pathology after vaccination and challenge with SARS-CoV-2. Hamsters were vaccinated intramuscularly (IM) or intranasally (IN) 10 (D10) or 28 (D28) days before IN challenge with SARS-CoV-2 Alpha, Beta, and Delta (D28 only) variants of concern. At 4 days post challenge, lung samples were collected and stained with H&E (200×). Hamsters receiving the control vaccine demonstrate moderate broncho-interstitial pneumonia and alveolar exudate regardless of the challenge variant **(A–E)**. No or minimal interstitial pneumonia was observed in IN-vaccinated hamsters **(F–J)**. Minimal to mild interstitial pneumonia was observed in IM-vaccinated hamsters **(K–O)**.

We sought to investigate the protective efficacy of our vaccine using two different routes of administration and 28 or 10 days between vaccination and challenge, respectively. Previous work in our lab demonstrated superior efficacy of IN vaccination in hamsters (21, 27), however, IM vaccination was more effective in NHPs when 10 days between vaccination and challenge were investigated (24). Here, we vaccinated hamsters with the VSV-SARS2-N+S vaccine at 10 or 28 days before challenge with the SARS-CoV-2 Alpha, Beta, or Delta VOC. All hamsters were euthanized 4 days post-challenge to determine lung pathology and viral loads. Protective efficacy was initially indicated by the lack of gross lung lesions and only observed in the IN-vaccinated groups compared to the control and IM-vaccinated cohorts of all three VOC (**Figure S2**). In general, IN vaccination with VSV-SARS2-N+S 10 or 28 days before challenge provided superior protection from interstitial pneumonia compared to IM vaccination or the control hamsters, regardless of challenge virus. Viral challenge with the Alpha variant at both 10- and 28-days post-vaccination (DPV) resulted in minimal interstitial pneumonia with rare foci of cellular spillover into alveolar spaces and minimal thickening of adjacent septa (**Figures 1F, H**). Similar results were observed at both vaccination timepoints for hamsters challenged with the Beta variant (**Figures 1G, I**). Moderate interstitial pneumonia was observed in only 17% (n=1/6) of Beta variant challenged hamsters in the 10 DPV IN group (**Figures 1F–I**). When comparing the vaccination-to-challenge interval between Alpha and Beta variants, IN challenge at 28 DPV resulted in no histopathologic lesions in 50% (n=3/6) for Alpha and 67% (n=4/6) for Beta variants. In contrast, no histopathologic lesions were observed in only 18% (n=1/6) of Alpha variant challenged hamsters in the 10 DPV IN group and 0% (n=0/6) for the Beta variant. These data suggest that 28 days are needed for the development of cross-variant protective immunity. Therefore, this timepoint only was assessed for protective efficacy for the Delta variant. We found that 5/6 Delta-challenged hamsters had no observable interstitial pneumonia with only one hamster in the group presenting with mild interstitial pneumonia (**Figure 1J**).

Challenge 10 or 28 DPV of the IM VSV-SARS2-N+S groups resulted in no subjective decrease in pulmonary inflammation at either timepoints relative to the VSV-EBOV vaccine control (**Figures 1A–E, K–O**). Alpha variant challenge 10 DPV by the IM route resulted in moderate interstitial pneumonia in (n=4/6) hamsters (**Figure 1K**). These results were enhanced after Beta variant challenge resulting in moderate (n=3/6) or severe (n=3/6) interstitial pneumonia (**Figure 1L**). Similarly, Alpha variant challenge 28 DPV by the IM route resulted in moderate (n=1/6) or severe (n=2/6) interstitial pneumonia (**Figure 1M**) while challenge with the Beta variant resulted in 100% (n=6/6) of hamsters exhibiting moderate interstitial pneumonia (**Figure 1N**). Similarly, 83% (n=5/6) of IM-vaccinated hamsters challenged with the Delta variant at 28 DPV exhibited moderate interstitial pneumonia (**Figure 1O**).

**Figure 2:**
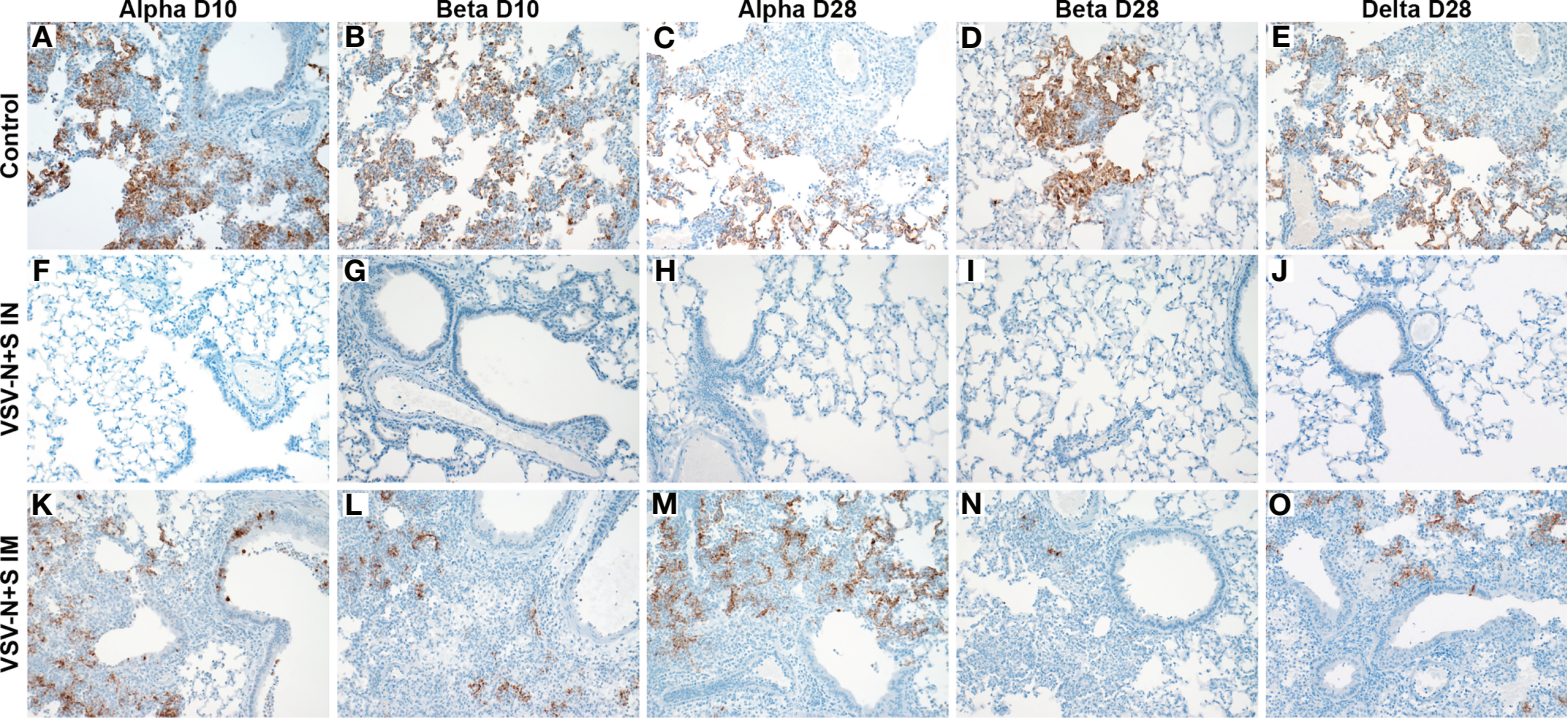
Immunohistochemistry of hamster lungs after vaccination and challenge with SARS-CoV-2 VOC. Hamsters were vaccinated intramuscularly (IM) or intranasally (IN) 10 or 28 days prior to IN challenge with SARS-CoV-2 Alpha, Beta, and Delta (D28 only) variants of concern. At 4 days post challenge, lung samples were collected and stained with anti-SARS-CoV-2 nucleocapsid antibody (200×). Hamster receiving the control vaccine demonstrate robust antigen staining regardless of the challenge variant **(A–E)**. Antigens were not observed in IN vaccinated hamsters **(F–J)**. Antigen immunoreactivity was observed in IM-vaccinated hamsters to a lesser extent than control animals **(K–O)**.

Immunohistochemistry revealed no SARS-CoV-2 antigen detection in the bronchiolar epithelium, type I and II pneumocytes, pulmonary macrophages and tracheal epithelium in any IN-vaccinated hamsters with the exception of the 10 DPV Beta challenge group (**Figures 2F–J**). However, in the 10 DPV Beta challenge group, rare immunoreactivity was observed in individual bronchiolar epithelial cells with no observable immunoreactivity lower in the respiratory tree (not shown). Disseminated immunoreactivity was observed throughout the pulmonary tree at every level immediately adjacent to, or within foci of interstitial pneumonia for all VSV-SARS2-N+S IM-vaccinated hamsters as well as VSV-EBOV-vaccinated hamsters (**Figures 2A–E, K–O**).

### Vaccination diminishes oral shedding and lung viral burden

**Figure 3:**
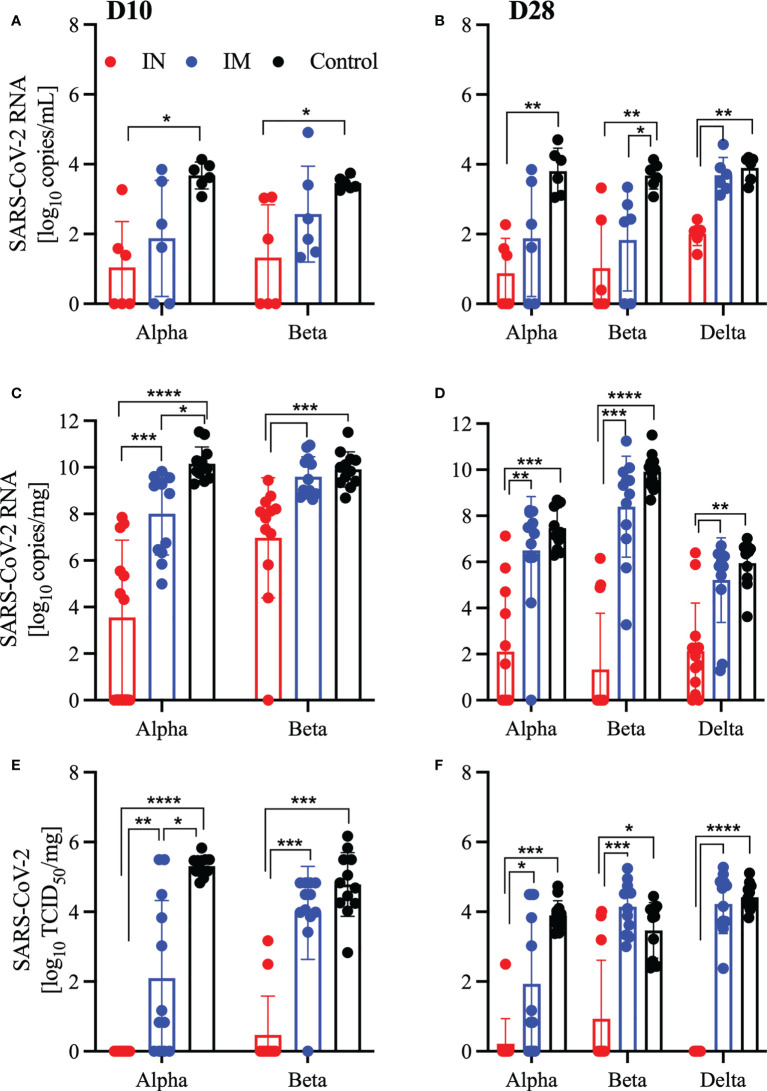
Vaccination reduces viral burden in oral swabs and lung samples. Hamsters were vaccinated intramuscularly (IM) or intranasally (IN) 10 or 28 days prior to IN challenge with SARS-CoV-2 Alpha, Beta, and Delta variants of concern. At 4 days post challenge, oral swab and lung samples were collected. SARS-CoV-2 viral RNA levels in oral swabs **(A, B)** and lung samples **(C, D)**. Infectious viral titer in hamster lungs were determined as median tissue culture infectious dose (TCID_50_) **(E, F)**. Geometric mean and geometric SD are depicted. Statistical significance as determined by the Mann–Whitney test is indicated as *p* < 0.0001 (****), *p* < 0.001 (***), *p* < 0.01 (**), and *p* < 0.05 (*).

An effective SARS-CoV-2 vaccine should control viral replication in the upper respiratory tract to reduce transmission and limit virus-induced lung damage. We investigated the ability of our vaccine to accomplish these goals through quantification of viral RNA in oral swabs and in the lungs of challenged hamsters. We found that across all challenge viruses and vaccination time points, IN vaccination significantly reduced viral RNA in the oral swabs compared to the controls (**Figures 3A, B**). In comparison, only the IM-vaccinated hamsters challenged 28 DPV with the Beta variant had a significant reduction in viral RNA compared to controls (**Figures 3A, B**). Next, we investigated whether the vaccine limited viral replication in the lungs and found that all IN-vaccinated hamsters presented with reduced viral RNA in the lungs compared to controls and IM-vaccinated hamsters (**Figures 3C, D**). This difference was confirmed by the infectious titer determination with all IN-vaccinated hamsters demonstrating significantly less infectious virus in the lungs compared to the controls and IM-vaccinated hamsters (**Figures 3E, F**). In contrast, IM-vaccinated hamsters presented with similar lung viral RNA loads and titers with the exception of the 10 DPV IM group challenged with the Alpha variant (**Figures 3C–F**).

### Antigen-specific IgG responses and their functionality following vaccination

**Figure 4:**
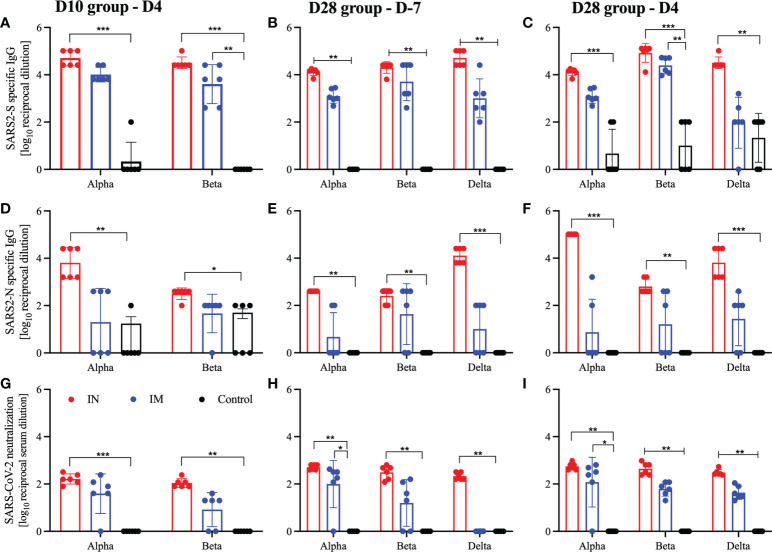
Humoral responses after viral challenge. Hamsters were vaccinated intramuscularly (IM) or intranasally (IN) 10 or 28 days before IN challenge with SARS-CoV-2 Alpha, Beta, and Delta variants of concern. At 7 days prior to challenge (D-7) or 4 days post-challenge (D4) serum samples were collected for analysis. **(A–C)** Levels of SARS-CoV-2 spike (S)-specific IgG; **(D–F)** levels of SARS-CoV-2 nucleocapsid (N)-specific IgG. Neutralization against homologous virus **(G–I)**. Geometric mean and geometric SD are depicted. Statistical significance as determined by the Mann–Whitney test is indicated as *p* < 0.001 (***), *p* < 0.01 (**), and *p* < 0.05 (*).

We measured the S- and N-specific humoral response at the time of euthanasia and found that only IN-vaccinated hamsters generated a consistent robust humoral response to either the S or N antigen, which were significantly higher than the control cohort (**Figures 4A–F**). While there were substantial antigenic IgG responses in IM-vaccinated hamsters, significance in the 10 DPV cohort was only achieved with the S antigen in the Beta-challenged animals (**Figure 4A**). There were significantly higher S-specific IgG titers for all groups 21 days after vaccination (D-7) in the D28 cohort, but on D4 after challenge only the Beta-challenged animals remained significant for IM-vaccinated hamsters (**Figures 4B, C**). A similar trend continued in the functional analysis of the humoral response; IN-vaccinated hamsters generated significantly more neutralizing antibodies at 10, 21, and 28 DPV regardless of the challenge variant. Conversely, IM-vaccinated hamsters produced neutralizing antibodies, 28 DPV for all viral variants and for the Alpha variant cohort 21 DPV (**Figures 4G–I**). We also assessed the cross-neutralization capacity of the serum from these vaccinated and challenged hamsters against Omicron BA.2. We found limited to no cross-reactive neutralizing antibodies in any of the groups tested (**Figure S3**).

## Discussion

For effective and sustained protection from COVID-19 a vaccine must have durability and cross-reactivity against newly emerging VOC. The vaccine-mediated immunity not only has to protect the lower respiratory tract against viral-induced damage, it also should reduce viral shedding. In addition, the immune response must be broad enough to remain effective against newly emerging viral variants. In this study, we demonstrated that a dual-antigen VSV-based vaccine is protective from disease caused by multiple SARS-CoV-2 VOC when administered IN in a single dose 28 and 10 days prior to viral exposure. All IN-vaccinated hamsters showed drastically reduced or absent gross and histopathologic lung lesions. The observed immunoreactivity is caused by the challenge virus not the vaccine virus, as VSV-based vaccines are highly susceptible to type I interferons and quickly cleared (21). IN-vaccinated hamsters presented with decreased viral RNA and infectious viral titers in the lungs. IN vaccination significantly reduced the viral RNA in the oral swabs suggesting a reduction in viral shedding. Due to the limited availability of reagents for hamsters, cellular phenotyping remains problematic in this model, however, humoral responses indicated that IN-vaccinated hamsters had significantly higher antigen-specific IgG and neutralization titers correlating with protection. As stated before, there are limitations to the cross-neutralization capacity of this vaccine as the antigen sequences are based on the ancestral strain. While the cross-neutralization potential directed against the S protein of “older” VOC is acceptable, the “newer” VOC Omicron BA.2 demonstrated limited cross-neutralization potential. Similar to vaccines based on other platforms currently in use, as the circulating virus diverges from the original sequence vaccines need to be updated to remain efficacious. Taken together, the data suggests that this dual-antigen VSV-based vaccine prevents severe disease associated with SARS-CoV-2 VOC in the hamster model. The reduction of oral viral RNA indicates a decrease in viral shedding which would decrease the potential of transmission.

The success of the COVID-19 vaccine development and implementation is on an unprecedented scale; SARS-CoV-2 is likely to become an endemic viral pathogen which will continue to adapt and evolve into new viral variants. With this emergence of new viral variants, the reduction of vaccine-elicited responses including neutralizing antibodies may continue (28). Using a vaccine that elicits a strong humoral response as well as a functional cellular response will greatly expand upon vaccine efficacy and durability even in the face of continued emerging VOC. In addition, the N protein retains high homology between viruses in the *Coronaviridae* family in part due to its importance for viral replication, highlighting its suitability as a vaccine antigen. In contrast, the S protein presents with increased variability as variants emerge (29–31). Immunoinformatic analysis and TCR epitope scanning have identified three T cell epitopes within the N protein that produce not only a strong polyfunctional CD8+ response but are also cross-reactive to other human coronaviruses (SPR, LPR, and PPK within the N_105-265_ region) (32–37). This response is dependent on the HLA phenotype of the individual. The most common HLA phenotype in the world, HLA-A2^+^, exhibits moderate polyfunctional CD8+ T cell response while HLA-B7^+^ has as strong reactivity when stimulated with the conserved epitopes (15, 38–40). The presence of strong cross-reactive epitopes paired with a person’s HLA phenotype could increase not only specific protection, but also a broader cellular polyfunctional response which will aid in cross-variant protection. Based on this rational, we incorporated the N antigen to more fully recruit the polyfunctional capabilities of the cellular immunity. Due to the limitations of the reagents available for the hamster model, we were not able to measure cellular activation phenotypes to determine if the inclusion of a strong T cell antigen contributed to protection. If the reagents become available, it would be of future interest of our lab to investigate those immunologic responses. In addition, mouse vaccine studies are underway to investigate the T cell contribution and durability of vaccine protection in another small animal model as vaccine efficacy likely wanes over time.

The generation of an immune response with the potential to inhibit the transmission of SARS-CoV-2 is essential for the reduction of case numbers and spread of this virus (41, 42). Due to the rapid burst of SARS-CoV-2 replication early in infection in the upper respiratory tract the existing immunologic environment can very easily become overwhelmed (43, 44). The systemic antibody response is less permissive to the upper respiratory tract, highlighting the importance of an antigen-specific, localized mucosal response for inhibition and clearance of the virus. Using previously published data for VSV-SARS2-S (27) comparing the reduction of viral shedding in hamsters challenged 28 DPV IN with our VSV-SARS2 N+S data, we demonstrate a significantly higher reduction of viral shedding in hamsters vaccinated with VSV-SARS2-N+S when challenged with the Alpha and Delta VOC (**Figure S4**). We hypothesize that the difference in tissue-resident memory T cells, specifically CD8+ T cells, have the ability to strongly inhibit viral infection (45) and may contribute to viral clearance from the upper respiratory tract if present. This hypothesis will be a topic of further research when the reagents become available to comprehensively characterize the resident T cell population of the nasal cavity after vaccination and challenge in hamsters.

The translational capacity of this vaccine can be highly related to the Merck derived VSVΔG-SARS-CoV-2 #9 (called V590) vaccine. Initially V590 followed the template of the approved VSV-EBOV vaccine and was administered IM. This resulted in low immunogenicity in a phase 1 clinical trial (46). Upon further evaluation in small animals models it was determined that the optimal route of vaccination was IN administration. This resulted in controlled viral replication not only in the lungs but also the upper respiratory tract (47). Those results mirror the results we demonstrated previously with our VSV-based SARS-CoV-2 vaccine constructs as well as VSV-SARS2-N+S here (21, 27). Seemingly, a mucosal route of vaccination results in a superior immune response, which is likely explained by the differences in the expression of the ACE2 receptor at the sites of vaccination. ACE2 is abundantly expressed in the upper respiratory tract and not the muscle, making the upper respiratory tract a more suitable environment for S-dependent viral replication which the VSV-SARS2-S and VSV-SARS2-N+S vaccines rely on (48–50).

In summary, we generated a single-dose dual-antigen vaccine with efficacy against multiple SARS-CoV-2 VOC. As we have demonstrated previously, the optimal route of vaccination is IN, with strong efficacy against challenge at 28 and 10 DPV. The fast-acting potential and hypothesized simultaneous stimulation of both the humoral and cellular immune response makes the dual-antigen VSV vaccine presented here an alternative booster vaccine against COVID-19. Future studies are planned to address whether existing SARS-CoV-2 immunity would impact the efficacy of the VSV-SARS2-N+S, both by vaccine induced immunity and prior viral challenge. Finally, the dual-antigen format would allow for a boost of the existing immunity generated by previously approved vaccines and allow for the diversification of cellular immunity to the secondary antigen potentially increasing the durability of the booster response.

## Data availability statement

The original contributions presented in the study are included in the article/**Supplementary Material**. Further inquiries can be directed to the corresponding author.

## Ethics statement

The animal study was reviewed and approved by Rocky Mountain Laboratories Animal Care and Use Committee.

## Author contributions

AM conceived the idea and secured funding. KLO and AM designed the studies. TG recovered the vaccine from plasmid and performed *in vitro* characterization. KLO, CSC and AM conducted the hamster studies. KLO, TG, PF, and CSC processed the samples and acquired the data. KLO, PF, CSC and AM analyzed and interpreted the data. KLO and AM prepared the manuscript with input from all authors. All authors have read and agreed to the published version of the manuscript.

## Funding

The study was funded by the Intramural Research Program, NIAID, NIH.

## Acknowledgments

We thank members of the Rocky Mountain Veterinary Branch, NIAID for supporting the hamster studies. We are grateful to Anita Mora and Austin Athman (NIAID) for assistance generating the pathology figures. The authors also thank members of the Molecular Pathogenesis Unit, Virus Ecology Section, and Research Technology Branch (NIAID) for their efforts to obtain and characterize the SARS-CoV-2 isolates used for challenge.

## Conflict of interest

The authors declare that the research was conducted in the absence of any commercial or financial relationships that could be construed as a potential conflict of interest.

## Publisher’s note

All claims expressed in this article are solely those of the authors and do not necessarily represent those of their affiliated organizations, or those of the publisher, the editors and the reviewers. Any product that may be evaluated in this article, or claim that may be made by its manufacturer, is not guaranteed or endorsed by the publisher.
