# Supplementary material for: Protection from COVID-19 with a VSV-based vaccine expressing the spike and nucleocapsid proteins
Source: Front Immunol. 2022 Oct 24;13:1025500. doi: 10.3389/fimmu.2022.1025500 (PMC9638159; doi:10.3389/fimmu.2022.1025500)
Supplement: Supplementary file 1 [file DataSheet_1.pdf]

Figure S1

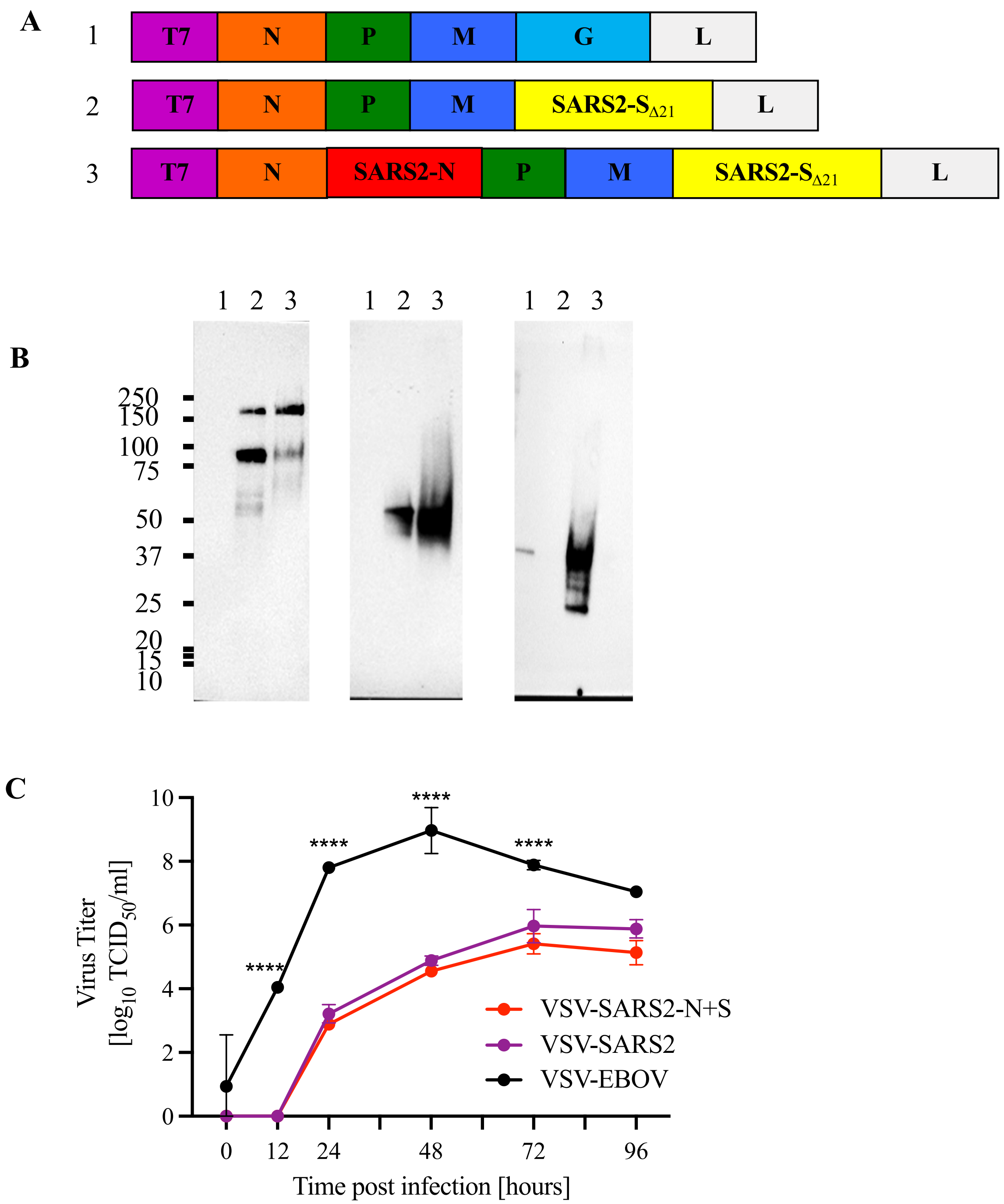

Figure S1. Schematic and characterization of VSV-based vaccines. (A) Schematic illustrating vaccine vector design. T7 promotor; N nucleoprotein; P phosphoprotein; M matrix protein; L RNA-dependent RNA polymerase; SARS-CoV-2 N; SARS-CoV-2 S. **(B)** Western blot analysis of cell supernatant/cell lysate samples containing VSV vaccines probed for SARS-CoV-2 S (left, cell supernatant), SARS-CoV-2 N (middle, cell lysate) or VSV-M (right, cell supernatant). 1 VSV wildtype; 2 pCAGGS-SARS2 N; 3 VSV-N-S. **(C)** Viral growth kinetics on Vero E6 cells. Geometric mean and geometric SD are depicted. Statistical significance as determined by two-way ANOVA with Tukey's multiple comparison is indicated as $p<0.0001$ (****).

Figure S2

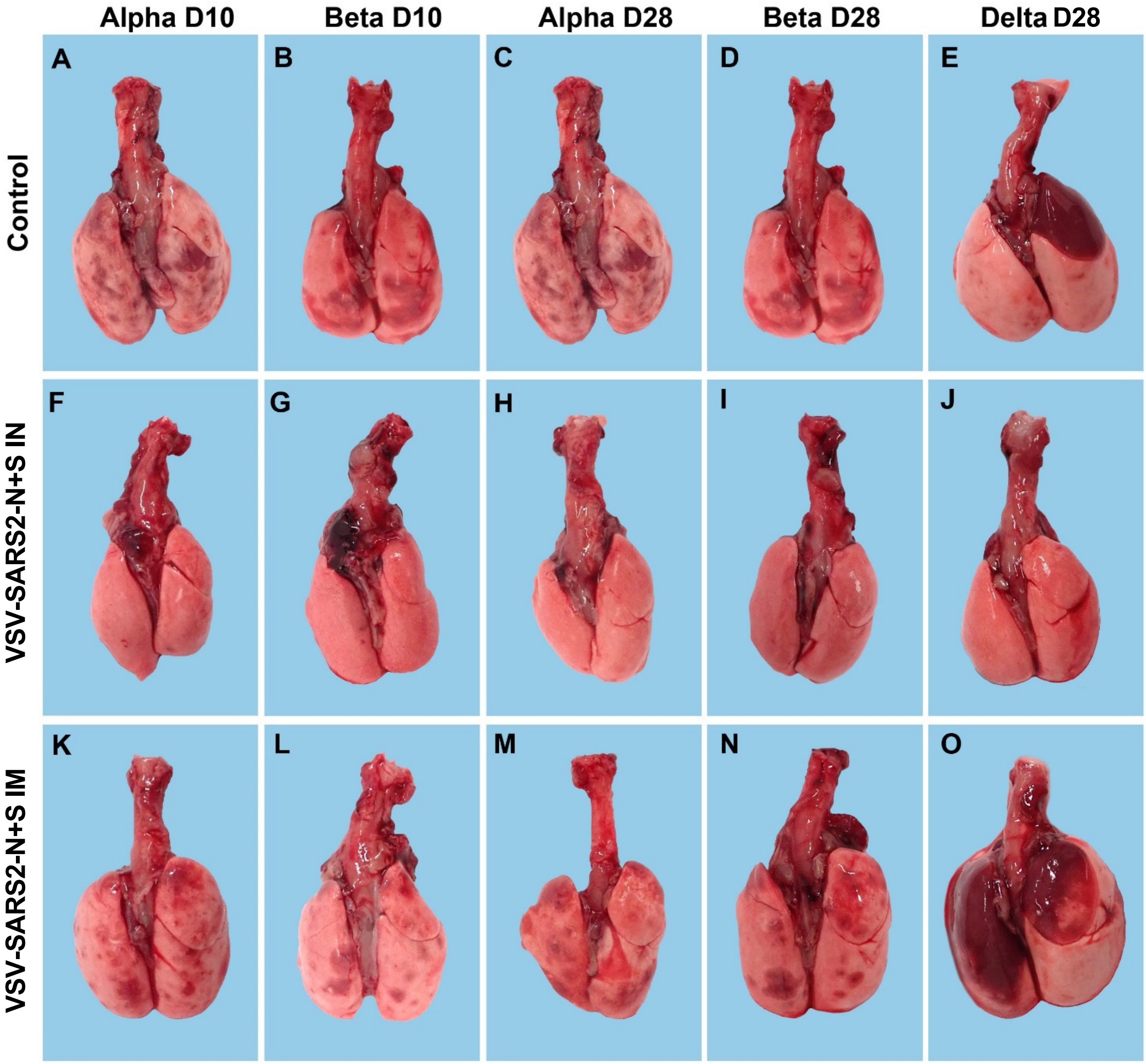

Figure S2. Hamster lung gross pathology after vaccination and challenge. Groups of hamsters were vaccinated with a single dose of the indicated vaccine by either the intramuscular (IM) or intranasal (IN) route and challenged with either the Alpha, Beta, or Delta (D28 only) variant as indicated 10 (D10) or 28 (D28) days later. Control animals **(A-E)**; IN-vaccinated animals **(F-J)**; IM-vaccinated animals **(K-O)**. Representative pictures of hamster lungs with lesions for each vaccine group at 4 days post-challenge.

Figure S3

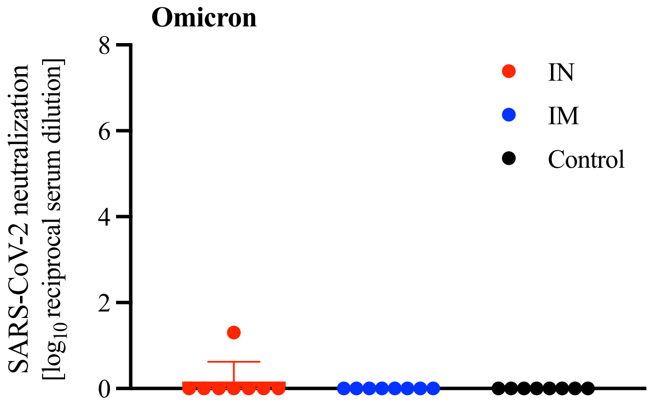

Figure S3. Cross-reactive humoral immune responses after viral challenge. Hamsters were vaccinated intramuscularly (IM) or intranasally (IN) 28 days before IN challenge with SARS-CoV-2 Alpha, Beta, and Delta variants of concern. 4 days post challenge serum samples were collected and pooled for neutralization against heterologous virus Omicron. Geometric mean and geometric SD are depicted.

Figure S4

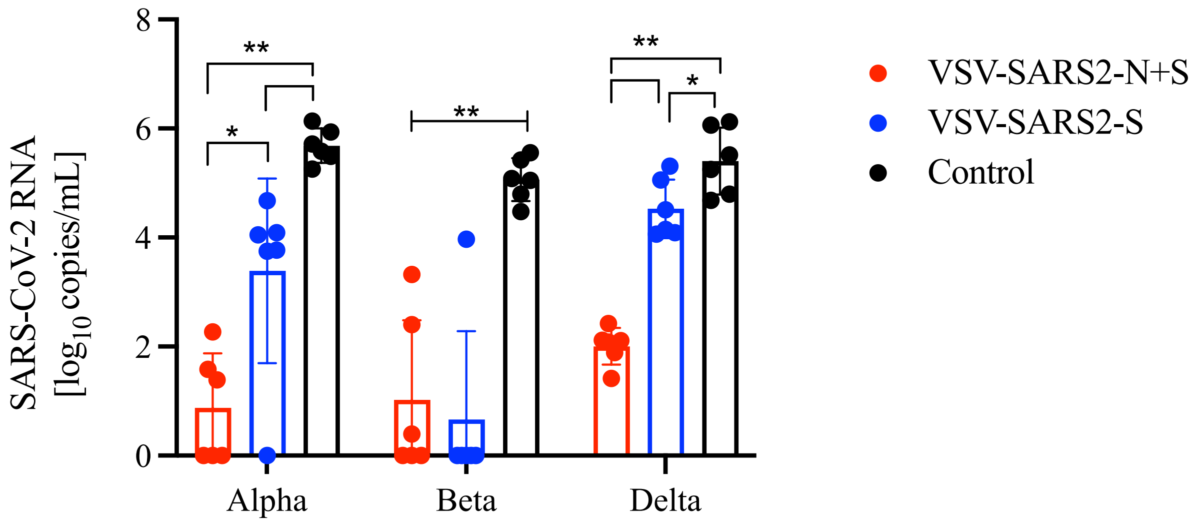

Figure S4. Comparison of reduction of viral shedding between animals vaccinated with VSV-SARS2-N+S, VSV-SARS2-S, and Control. Hamsters were vaccinated intranasally (IN) 28 days before IN challenge with SARS-CoV-2 Alpha, Beta, and Delta variants of concern. 4 days post challenge oral swab samples were collected and analyzed by rt-qPCR to determine the reduction in viral shedding. Geometric mean and geometric SD are depicted. Statistical significance as determined by the Mann–Whitney test is indicated as $p < 0.01$ (**), and $p < 0.05$ (*).
